# Supplementary material for: Unveiling the S=3/2 Kitaev honeycomb spin liquids
Source: Nat Commun. 2022 Jul 2;13:3813. doi: 10.1038/s41467-022-31503-0 (PMC9250503; doi:10.1038/s41467-022-31503-0)
Supplement: Supplementary file 1 — Supplementary Information [file 41467_2022_31503_MOESM1_ESM.pdf]

Supplementary Information for "Unveiling the S=3/2 Kitaev Honeycomb Spin Liquids"

Hui-Ke Jin[1], W. M. H. Natori[2,3], F. Pollmann[4,5], and J. Knolle[1,3,5]

[1]Department of Physics TQM, Technische Universität München,
James-Franck-Straße 1, D-85748 Garching, Germany
[2]Institute Laue-Langevin, BP 156, 41 Avenue des Martyrs, 38042 Grenoble Cedex 9, France
[3]Blackett Laboratory, Imperial College London, London SW7 2AZ, United Kingdom
[4]Department of Physics CMT, Technische Universität München,
James-Franck-Straße 1, D-85748 Garching, Germany
[5]Munich Center for Quantum Science and Technology (MCQST), 80799 Munich, Germany

June 14, 2022

In this Supplementary Information, we show more details about (i) the self-consistent mean-field theory for S=3/2 Kitaev honeycomb model and (ii) the spin quadrupolar parameter.

Supplementary Note 1: Self-consistent mean-field theory

In the main text, we have introduced a mean-field Hamiltonian $H(\{u\}=1)$ with the following mean-field order parameters:

$$
\begin{aligned}
Q_i^{ab} &= -\left\langle i\theta_i^a\theta_i^b\right\rangle \quad (a \neq b), \\
\Delta_{ij}^{ab} &= \left\langle i\theta_i^a\theta_j^b\right\rangle .
\end{aligned} \tag{1}
$$

For concreteness, we investigated the mean-field theory of the model with fixed exchange parameters $J_x = J_y = 1$ and varying J_z and D_z. In this parameter regime, the model preserves

mirror symmetry M_z, inversion symmetry I, and time-reversal symmetry $\mathcal{T}$ (see Supplementary Fig. 1), which will impose constraints to the order parameters and allow us to provide a succinct form for the mean-field Hamiltonian $H(\{u\} = 1)$. In accordance with a full symmetry analysis [1], we find that for a translational invariant solution which preserves M_z, I, and $\mathcal{T}$ symmetries there exist only eight non-zero and independent mean-field order parameters

$$Q^{xy}, \Delta_x^{zx}, \text{ and } \Delta_{z(x)}^{aa}, \ (a = x, y, z),$$

where the bond parameters $\Delta_{x(z)}^{ab}$ are defined on the x-type (z-type) bonds.

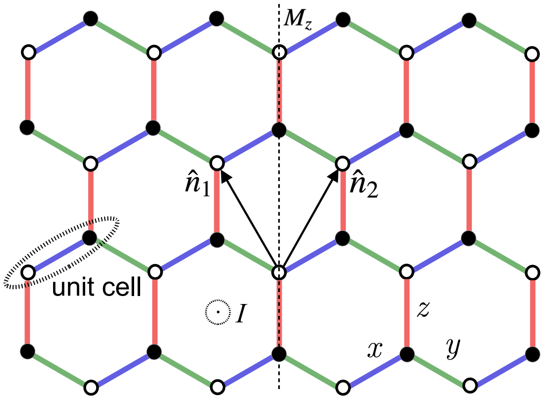

Supplementary Figure 1: The mirror symmetry M_z and inversion symmetries I for the Kitaev model on a honeycomb lattice. $\hat{n}_1$ and $\hat{n}_2$ are two Bravais lattice vectors. The dots and circles denote the A and B sublattices, respectively.

We can write down the mean-field Hamiltonian $H_{\mathrm{MF}}(\{u\} = 1)$ in the reciprocal space as

$$H_{\mathrm{MF}}(\{u\} = 1) = \sum_{\mathbf{k}} \psi_{\mathbf{k}}^{\dagger} H_{\mathbf{k}} \psi_{\mathbf{k}}, \quad \psi_{\mathbf{k}}^{\dagger} = \left(\theta_{A,\mathbf{k}}^{z}, \theta_{A,\mathbf{k}}^{x}, \theta_{A,\mathbf{k}}^{y}, \theta_{B,\mathbf{k}}^{z}, \theta_{B,\mathbf{k}}^{x}, \theta_{B,\mathbf{k}}^{y}\right) \tag{2}$$

where the Fourier transformed fermions

$$\theta_{A(B),\mathbf{k}}^{a} = \frac{1}{\sqrt{N}} \sum_{\mathbf{r}} e^{-i\mathbf{k}\cdot\boldsymbol{r}} \theta_{A(B),\mathbf{r}}^{a}, \quad \left(\theta_{A(B),\mathbf{k}}^{a}\right)^{\dagger} = \theta_{A(B),-\mathbf{k}}^{a}$$

on the A (B) sublattice are complex fermions rather than Majorana fermions, and $\mathbf{r}$ denotes the

Phase	(J_z, D_z)	Q^{xy}	Δ_z^{zz}	Δ_z^{xx}	Δ_z^{yy}	Δ_x^{zz}	Δ_x^{xx}	Δ_x^{yy}	Δ_x^{zx}
Isotropic	$(1, 0)$	0	0.760	-0.093	0.525	0.120	0.547	0.525	-0.369
A_0	$(0.6, 0)$	-0.633	0.150	-0.0016	0.057	0.540	0.381	-0.458	-0.390
A_0	$(0.2, 0)$	-0.644	0.060	-0.0005	0.025	0.550	0.373	-0.455	-0.390
B	$(1.2, 0)$	0.891	0.9997	-4×10^{-5}	5×10^{-5}	0.005	0.316	-0.316	-0.011
B	$(1.6, 0)$	0.914	0.9999	-1×10^{-5}	2×10^{-5}	0.003	0.283	-0.283	-0.007
A_0	$(1, 1)$	-0.860	0.1885	-0.0014	0.0349	0.5691	0.2146	-0.2627	-0.2886
A_z	$(8, 4)$	-0.949	0.8365	-8×10^{-4}	1.5×10^{-3}	0.2377	0.1395	-0.1404	-0.1714
B	$(4, 1)$	0.9663	$1 - 4 \times 10^{-6}$	-3×10^{-7}	3×10^{-7}	0.00048	0.1813	-0.1813	-0.0014
A_0	$(1, \infty)$	-1	0.1807	0	0	0.627	0	0	0
A_0	$(4, \infty)$	-1	0.5248	0	0	0.5248	0	0	0
A_z	$(8, \infty)$	-1	0.9104	0	0	0.2155	0	0	0

Supplementary Table 1: The self-consistent solutions for some typical values of J_z and D_z. Here $J_x = J_y = 1$ and a zero-flux configuration of $\{u\} = 1$ is chosen. Notice that the self-consistent solution for effective isotropic S=1/2 Kitaev spin liquid at $(J_z = 4, D_z \to \infty)$ is equivalent to that given in Ref. [3] up to a factor of -2, where the minus sign is caused by the antiferromagnetic couplings $J_a > 0$ and the factor of 2 is caused by the normalization condition of Majorana fermions used here, *e.g.*, $(\theta_i^a)^2 = (\eta_i^a)^2 = 1$.

unit cell coordinates. Then, the self-consistent equations can be written as

$$Q^{ab} = \frac{1}{N} \sum_{\mathbf{k}} - \left\langle i\theta^a_{A(B),\mathbf{k}} \theta^b_{A(B),-\mathbf{k}} \right\rangle , \tag{3a}$$

$$\Delta_c^{ab} = \frac{1}{N} \sum_{\mathbf{k}} e^{-i\mathbf{k}\cdot\hat{n}_c} \left\langle i\theta^a_{A,\mathbf{k}} \theta^b_{B,-\mathbf{k}} \right\rangle , (c = x, y, z), \tag{3b}$$

where $\hat{n}_x = \vec{0}$, $\hat{n}_y = \hat{n}_1$, and $\hat{n}_z = \hat{n}_2$. The choice of unit cell and the definition of the Bravais lattice vectors $\hat{n}_{1(2)}$ are shown in Supplementary Fig. 1.

An iterative scheme has been employed to find the self-consistent solutions to the mean-field Hamiltonian introduced in the main text, where different randomly-generated parameters are selected to initialize the iterations. If multiple inequivalent solutions occurred, we will select the solution with the lowest MF ground-state energy. The self-consistent solutions for some typical values of J_z and D_z are shown in Supplementary Table 1. We also show the results of the mean-field parameter as a function of iterative steps in our iterative self-consistent calculations in

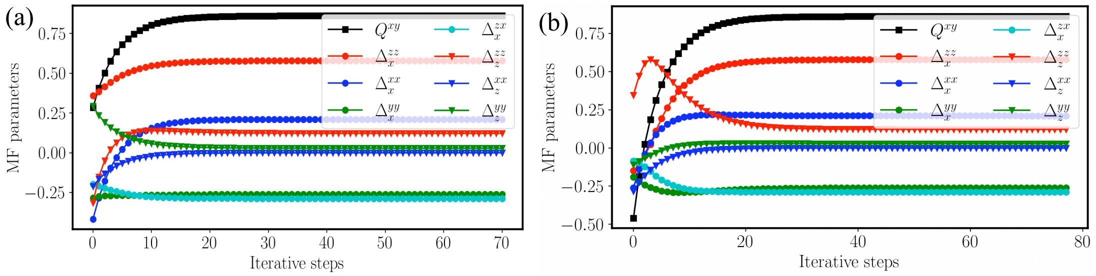

Supplementary Figure 2: The mean-field parameters as a function of iterative steps, where $J_x = J_y = 1$, $J_z = 0.6$ and $D_z = 1$. Different randomly generated parameters are selected to initialize the iterations in (a) and (b).

Supplementary Figs. 2(a) and (b), where two different randomly generated parameters converge to the same solution.

Supplementary Note 2: Spin quadrupolar parameter

The spin quadrupolar parameter, which is distinguished from magnetic order, is time-reversal invariant. This order usually does not exist in the $S = 1/2$ systems because a product of arbitrary two spin-1/2 operators is still a spin-1/2 operator or a trivial identity matrix. While for higher spin systems, the product of two spin operators gives rise to nontrivial spin quadrupolar operators which generally can support the spin quadrupolar parameters. A simple example of spin quadrupolar parameter for $S = 1$ systems can be found in Ref. [2].

The Z_2 quantum spin liquid state $|\Psi\rangle$ shown in the phase diagram in the main text generally coexists with a spin quadrupolar parameter, *e.g.*,

$$\begin{aligned} \langle\Psi|S_j^a|\Psi\rangle &= 0, \\ \langle\Psi|(S_j^x)^2 - (S_j^y)^2|\Psi\rangle &= 0, \\ \langle\Psi|(S_j^z)^2|\Psi\rangle - 5/4 &= Q^{xy}, \end{aligned} \tag{4}$$

where the last line in Eq. (4) is the basic definition of the spin quadrupolar parameter in the main text. Note that these properties in Eq. (4) cannot be resolved by a single product state.

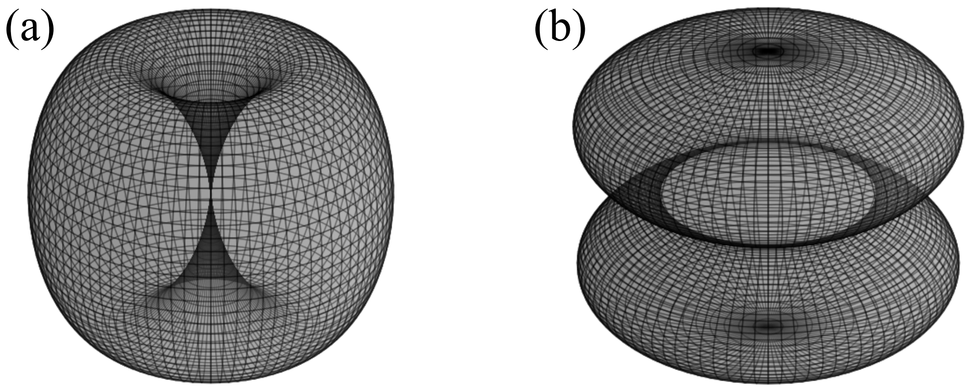

Supplementary Figure 3: Probabilities of spin fluctuations $P(Q^{xy}, \hat{n})$ [see definition in Eq. (5)] for (a) $Q^{xy} = -1$ and (b) $Q^{xy} = 1$.

The spin quadrupolar parameter usually is illustrated by the probabilities of spin fluctuations [2]. Since $|\Psi\rangle$ is a many-body state, for illustration purposes we utilize the probabilities of spin fluctuations between S=3/2 spin coherent states $|S(\hat{n})_j\rangle$ and the reduced density matrix $\rho_j(Q^{xy})$ for site j:

$$P\left(Q^{xy}, \hat{n}\right) = \mathrm{Tr}\left[|S_j(\hat{n})\rangle\langle S_j(\hat{n})|\, \rho_j(Q^{xy})\right], \tag{5}$$

where $|S_j(\hat{n})\rangle$ with $\left(\hat{n} \cdot \vec{S}_j\right) |S_j(\hat{n})\rangle = S|S_j(\hat{n})\rangle\ \hat{n}$ is a spin coherent state pointing to the direction of $\hat{n}$. The probabilities of spin fluctuations for $Q^{xy} = -1$ and $Q^{xy} = +1$ are shown in Supplementary Figs. 3(a) and (b), respectively. Here the reduced density matrix $\rho_j(Q^{xy})$, which can resolve Eq. (4), is obtained by exact diagonalization on a 2×2 torus.

Supplementary Reference

[1] *et al*, W. M. H. N. In preparation.

[2] Läuchli, A., Mila, F. & Penc, K. Quadrupolar phases of the $S = 1$ bilinear-biquadratic Heisenberg model on the triangular lattice. *Phys. Rev. Lett.* **97**, 087205 (2006).

[3] You, Y.-Z., Kimchi, I. & Vishwanath, A. Doping a spin-orbit Mott insulator: Topological superconductivity from the Kitaev-Heisenberg model and possible application to $(Na_2/Li_2)IrO_3$. *Phys. Rev. B* **86**,
